# Dr. L. P. Kennedy

**Published:** 1892-01

**Authors:** 


### DR. L. P. KENNEDY.

In our last issue we announced that Dr. Kennedy seemed on the road to recovery.

It pains us to have to announce that the doctor has since developed less favorable symptoms and his condition is now critical.

Dr. Kennedy has been one of the proprietors and editors of THE JOURNAL for the past two years, and his associates have had ample opportunity of proving his character as an honest, upright, sincere Christian gentleman.

His numerous friends, and his associates upon THE JOURNAL, hope to soon see him well again, and once more in the position which he so well filled.

